# Reactive Oxygen Species Modulate the Barrier Function of the Human Glomerular Endothelial Glycocalyx

**DOI:** 10.1371/journal.pone.0055852

**Published:** 2013-02-14

**Authors:** Anurag Singh, Raina D. Ramnath, Rebecca R. Foster, Emma C. Wylie, Vincent Fridén, Ishita Dasgupta, Borje Haraldsson, Gavin I. Welsh, Peter W. Mathieson, Simon C. Satchell

**Affiliations:** 1 Academic Renal Unit, Clinical Sciences North Bristol, Southmead Hospital, Bristol, United Kingdom; 2 Division of Medicine, Chilliwack General Hospital, Fraser Health Authority Chilliwack, British Columbia, Canada; 3 Renal Unit, Institute of Medicine, Sahlgrenska Academy, Gothenburg University, Gothenburg, Sweden; Rouen University Hospital, France

## Abstract

Reactive oxygen species (ROS) play a key role in the pathogenesis of proteinuria in glomerular diseases like diabetic nephropathy. Glomerular endothelial cell (GEnC) glycocalyx covers the luminal aspect of the glomerular capillary wall and makes an important contribution to the glomerular barrier. ROS are known to depolymerise glycosaminoglycan (GAG) chains of proteoglycans, which are crucial for the barrier function of GEnC glycocalyx. The aim of this study is to investigate the direct effects of ROS on the structure and function of GEnC glycocalyx using conditionally immortalised human GEnC. ROS were generated by exogenous hydrogen peroxide. Biosynthesis and cleavage of GAG chains was analyzed by radiolabelling (S^35^ and ^3^H-glucosamine). GAG chains were quantified on GEnC surface and in the cell supernatant using liquid chromatography and immunofluorescence techniques. Barrier properties were estimated by measuring trans-endothelial passage of albumin. ROS caused a significant loss of WGA lectin and heparan sulphate staining from the surface of GEnC. This lead to an increase in trans-endothelial albumin passage. The latter could be inhibited by catalase and superoxide dismutase. The effect of ROS on GEnC was not mediated via the GAG biosynthetic pathway. Quantification of radiolabelled GAG fractions in the supernatant confirmed that ROS directly caused shedding of HS GAG. This finding is clinically relevant and suggests a mechanism by which ROS may cause proteinuria in clinical conditions associated with high oxidative stress.

## Introduction

Cardiovascular disease remains one of the principle causes of mortality in both the developed and the developing worlds [1]. Scientific and clinical research in the last two decades has confirmed the associations between microalbuminuria, cardiovascular disease and progressive kidney disease [2]. This is particularly evident in systemic diseases like diabetes and hypertension where the onset of microalbuminuria strongly predicts death [3], [4]. Although, generalized damage to the vascular endothelium is believed to offer a plausible explanation of the link between the kidney and systemic circulation, the precise mechanisms of injury are yet to be elucidated [5].

Vascular oxidative stress is an important factor leading to endothelial dysfunction and has been identified as a significant contributor to the progression of atherosclerosis and other vascular complications of diabetes [6], [7,]. Excessive generation of reactive oxygen species (ROS) is central to the pathogenesis of diabetic nephropathy [8]. Proteinuria heralds the onset of kidney disease in diabetes. The wall of the glomerular capillary is the primary site of sieving action in the kidney and is known as the glomerular filtration barrier (GFB) [9]. The GFB is uniquely adapted to be ‘selectively permeable’ i.e. impermeable to macromolecules but allow relatively free passage of water and solutes and is very tightly regulated in health. This property of the GFB depends on the combined action of its components: the podocytes, glomerular basement membrane and the endothelium with its glycocalyx [10]. The roles of glomerular podocytes and the glomerular basement membrane in the pathogenesis of proteinuric kidney diseases have been described extensively. However, due to difficulties in isolation and lack of experimental models, the vital contribution of the glomerular endothelial cell (GEnC), and particularly its glycocalyx, has only recently been appreciated [11]–[14].

GEnC are highly specialized cells whose luminal surface is covered by a layer of glycocalyx 200–400 nm in thickness and which covers both fenestral and inter-fenestral domains [15], [16], [17]. The glycocalyx is a dynamic hydrated layer largely composed of proteoglycans, glycosaminoglycans (GAG) and adsorbed plasma proteins [14], [18]. Proteoglycans, particularly heparan sulphate (HS) proteoglycans are largely responsible for the anionic charge characteristics of the glycocalyx.

Vascular endothelial cells are vulnerable to damage by ROS and glycocalyx is believed to act as a ‘vasculo-protective shield’ [18]. Hence, endothelial glycocalyx is a major site of action of circulating ROS and cytokines produced during oxidative stress [19]–[21]. Intact endothelial glycocalyx also has binding sites for anti-oxidant enzymes like xanthine oxido-reductase [22] and endothelial superoxide dismutase (eSOD) [23] so, has its own capacity to quench free radicals. Therefore, loss of endothelial glycocalyx exposes the vascular endothelium to the deleterious effects of ROS, which subsequently leads to endothelial dysfunction.

These observations are relevant to the glomerular microcirculation particularly in understanding the pathogenesis of proteinuria in states of high oxidative states. Here we investigate the direct effects of exogenous ROS on the key components of human GEnC glycocalyx and quantify consequent changes in its protein restrictive barrier function. We test the hypotheses that exposure to ROS directly alters the critical components of the GEnC glycocalyx and that these alterations have implications for its barrier action to the passage of albumin.

## Results

### ROS Reduce Expression of WGA Lectin and HS GAG without any Deleterious Effect on Cell Survival

**Figure 1:**
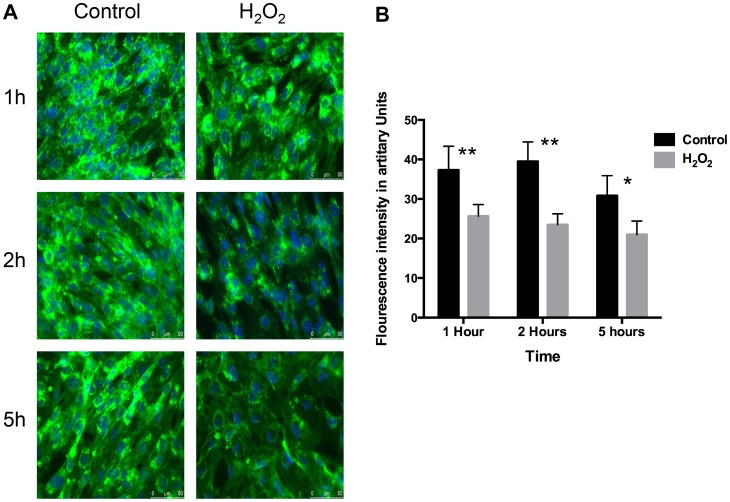
WGA lectin after ROS. A Fluorescence microscopy after labeling GEnC with WGA-FITC lectin and nuclear staining with DAPI. Left column: represents ‘control’ images (no treatment). Right column: represents images after treatment with100 µM of H_2_O_2_. The rows define the time periods: 1 h, 2 h and 5 h. These images show reduction in the binding of WGA-FITC lectin after treatment with H_2_O_2_ over time. **B** Bar chart showing quantitative comparisons of fluorescence intensity of WGA-FITC lectin between GEnC treated with vehicle only and H_2_O_2_ (100 µM) for 1 h, 2 h and 5 h. Fluorescence is quantified by using NIH Image J software. The chart shows significant reduction in the expression of WGA-FITC after treatment with H_2_O_2_ (n = 10, p = 0.01, ANOVA).

**Figure 2:**
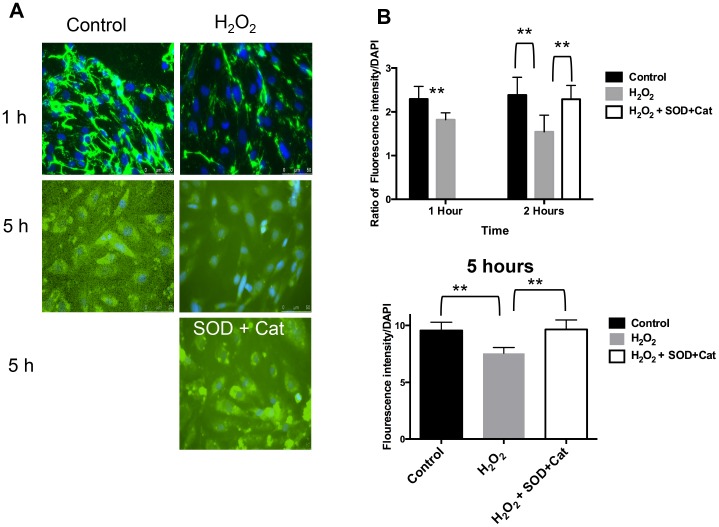
Expression of HS GAG on GEnC surface after ROS. A Immunofluorescence microscopy after staining GEnC with anti-HS antibody and nuclear staining with DAPI. Left column: represents ‘control’ images (no treatment). Right column: represents images after treatment with100 µM of H_2_O_2_. 1st and 2nd rows define the time periods: 1 h and 5 h. 3rd row: Image in the right column shows GEnC treated with both H_2_O_2_ and inhibitors, superoxide dismutase (SOD) and catalase (Cat). These images show reduction in anti-HS staining after treatment with H_2_O_2_ over time. This effect can be blocked in the presence of SOD and Cat. **B** Fluorescence intensity after immunostaining of GEnC with anti-HS antibody over time. Comparisons are shown between controls, H_2_O_2_ (100 µM) and H_2_O_2_ (100 µM)+SOD and Cat. Y-axis shows ratio of fluorescein emission (from HS) with nuclear staining (DAPI) which is used as a control for cell numbers. The top bar graph shows significant reduction in HS after 1 h and 2 h of H_2_O_2_ treatment (n = 11; p<0.001, t-test). Adding SOD and catalase at the same time as H_2_O_2_ blocks the effect of H_2_O_2_ analyzed at 2 h (n = 11; p<0.01, t-test). The lower bar graph confirms the effects of H_2_O_2_ can be reproduced at 5 h (n = 16; p<0.01, t-test).

GEnC monolayers exposed to H_2_O_2_ reveal a significantly reduced cell surface binding of WGA lectin compared to control over 1, 2 and 5 hours (Figure 1A). This is quantified by showing a significant reduction in the fluorescent intensity after treatment with H_2_O_2_ over 5 hours (Figure 1B). Similarly, there is significant reduction in the expression of cell surface HS after H_2_O_2_ treatment over time (Figure 2A & 2B). This effect of H_2_O_2_ on HS can be blocked if the cells are co-incubated with free radical scavengers, superoxide dismutase (SOD) and catalase (Cat) along with H_2_O_2_. The latter result confirms that the changes seen after H_2_O_2_ are specifically as a result of ROS.

**Figure 3:**
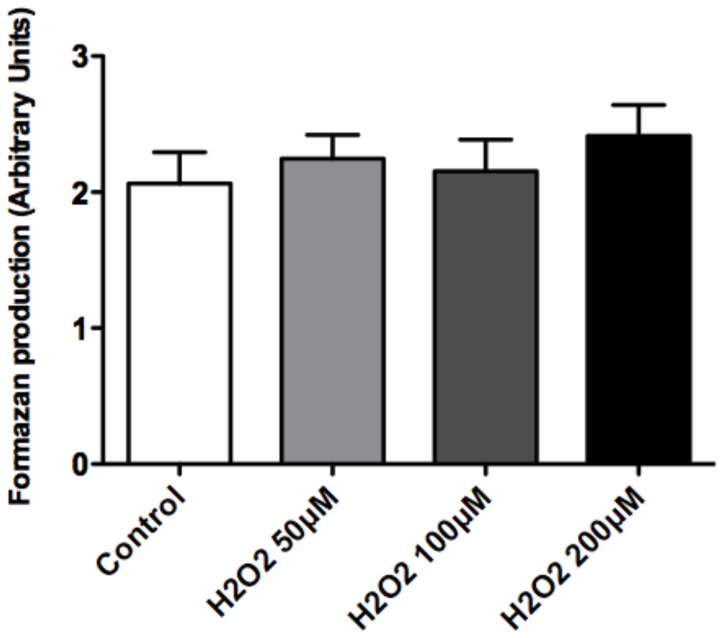
Cell survival assay after ROS. Bar graph showing measurement of absorbance in arbitrary units (Y axis), which estimates amount of formazan produced by GEnC which is used as a surrogate for cell survival. GEnC were treated with control (vehicle), 50, 100 and 200 µM of H_2_O_2_. Results show no significant differences (n = 12; p = ns; ANOVA).

We also tested the GEnC monolayers for any adverse cytotoxic effect of H_2_O_2_. Exposure to H_2_O_2_ does not affect the survival or morphology of GEnC at the concentrations of 50–200 µM (Figure 3).

### ROS Exposure does not Alter Biosynthesis of GAG Chains

**Figure 4:**
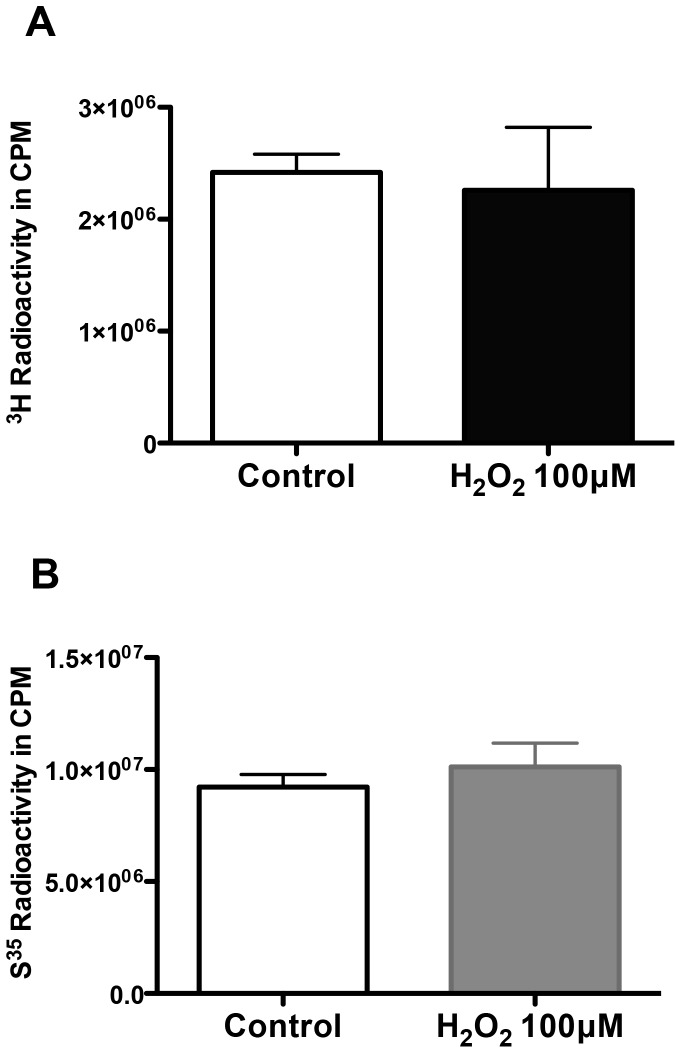
Biosynthesis of GAG chains after ROS. A&B Charts showing comparison of **A,** the cumulative 3H^3^-glucosamine or **B,** S^35^ labeled GAG incorporated into the GEnC under control versus H_2_O_2_ conditions. Y-axes represent amount of radioactivity in counts per minute (CPM). Results show no significant changes in the rate of biosynthesis of neither non-sulphated (3H^3^-glucosamine) nor sulphated GAG (S^35^) after exposing cells to H_2_O_2_ (n = 6 for both experiments; p = 0.12 & 0.19 respectively).

Study of the tritiated-glucosamine incorporated into the glycocalyx of GEnC (a measure of both sulphated and non-sulphated GAG chains, as glucosamine is a component of the disaccharide chain of GAG “backbone”) revealed that exposure to H_2_O_2_ for 48 h did not cause a significant change compared to controls. This is evident (Figure 4A) in the cumulative data of the isolated fractions. Incorporation of S^35^ was also not significantly changed by H_2_O_2_ (Figure 4B). These results imply the marked reduction in lectin binding and HS expression observed in the previous experiment is not a result of reduction in GAG biosynthesis.

### ROS Leads to Increased Cleavage of Sulphated GAG from GEnC Glycocalyx

**Figure 5:**
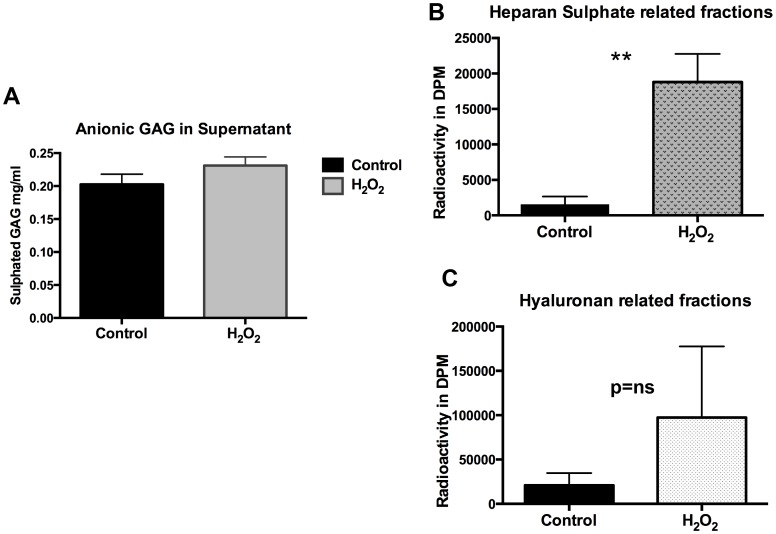
Estimation of GAG in GEnC supernatant. A Graph showing analysis of highly anionic GAG present in the supernatant following treatment of GEnC with controls versus H_2_O_2_. The experiment utilizes Alcian Blue dye that binds to the anionic residues of proteoglycans. Cumulative data from individual experiments shows significant increase in the quantity of alcian blue staining in the supernatant after H_2_O_2_. This confirms cleavage of GAG residues from the surface of GEnC after exposure to ROS (n = 3 experiments (individual experiment replicates = 8–12), p<0.05). **B** Chart showing comparison of 3H^3^-glucosamine labeled HS GAG fractions isolated from GEnC supernatant under control and post-H_2_O_2_ conditions. Results show marked increase in the HS GAG fractions in the supernatant compared to controls after treatment with H_2_O_2_ suggesting cleavage of HS GAG (n = 4; p = 0.0019; t test). **C** Chart showing comparison of 3H^3^-glucosamine labeled Hyaluronan GAG (non-sulphated) fractions isolated from GEnC supernatant under control and post-H_2_O_2_ conditions. Results show a trend but not a significant increase in the non sulphated/Hyaluronan GAG fractions in the supernatant compared to controls. (n = 4; p = 0.053; t test).

Following the results from the GAG biosynthesis experiment. We then wanted to quantify cleaved GAG residues in the GEnC supernatant after exposure to H_2_O_2_. This was done using two techniques: Alcian Blue assay and quantification of radiolabelled GAG fractions in the supernatant after exposure to H_2_O_2._ Alcian Blue has high affinity to sulphated GAG residues due to its high anionic density. Results show a significant increase in the Alcian Blue binding in the supernatant of the GEnC exposed to H_2_O_2_ compared to controls (Figure 5A) suggesting cleavage of GAG chains. Further, radiolabelling studies to characterize the GAG in supernatant show markedly elevated levels of fractions with HS (Figure 5B) rather than HA (Figure 5C) confirming the loss of highly anionic sulphated GAG. This result confirms that the apparent reduction in the expression of cell surface HS demonstrated on immunofluorescence is as a result of direct cleavage from the GEnC surface.

### ROS Transiently Reduce Trans-endothelial Electrical Resistance (TEER) of GEnC Monolayers

**Figure 6:**
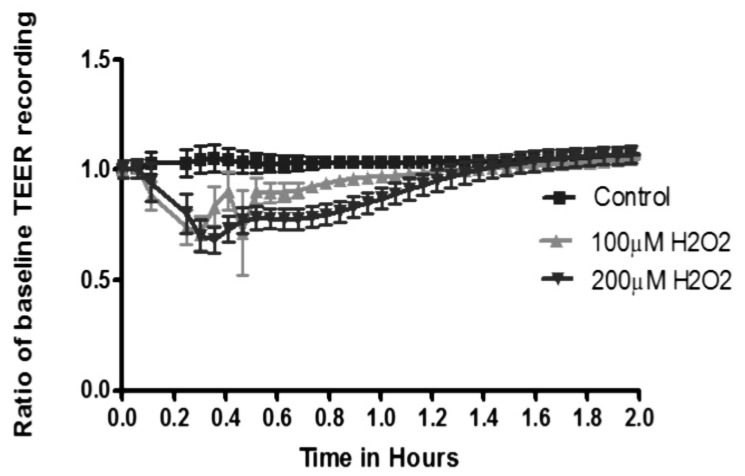
Transendothelial electrical resistance after ROS. Graph showing real time trans-endothelial electrical resistance (TEER) recordings of GEnC monolayers during exposure to H_2_O_2_. H_2_O_2_ is added at time point 0 and measurements are taken every 2 min. TEER (Y-axis) shown as a ratio of baseline recording versus time (X-axis). Results show a reduction in TEER after addition of H_2_O_2_ within the first 2 min. This effect is significant and peaks at 24 min followed by recovery by 60 min. There is complete resolution of changes in TEER by 90 min.

GEnC monolayers treated with H_2_O_2_ show an immediate, but non-sustained reduction in the TEER measured in a real time analyzer. H_2_O_2_ is added at time zero, showing significant effect in less than 2 min with maximal effect at 30 min showing reduction in TEER compared to control GEnC treated with vehicle only (Figure 6). This effect completely resolves by 90 min with TEER returning to baseline.

### ROS Cause a Significant and Sustained Increase in Passage of Albumin Across GEnC Monolayers

**Figure 7:**
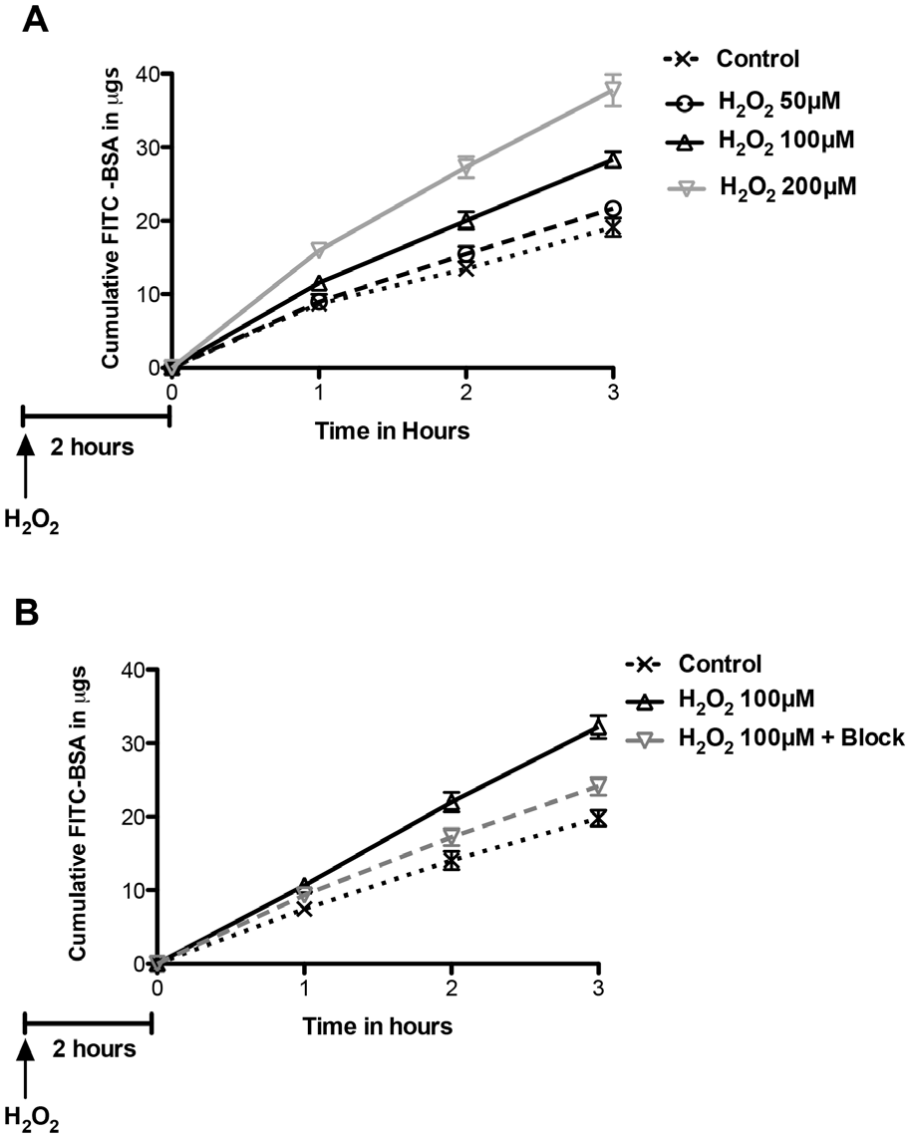
Changes in albumin passage after ROS. A Graph showing cumulative passage of fluorescein labeled albumin (Y-axis) across GEnC monolayers over time (X-axis). Analysis was performed after a delay of 120 min after addition of H_2_O_2_ to allow for recovery of TEER of GEnC monolayers. GEnC exposed to H_2_O_2_ show a dose dependant increase in passage of albumin compared to controls (n = 6, p<0.001 for effect of treatment, 2-way ANOVA) **B** Graph showing data from of a separate experiment confirming the significant increase in passage of labeled albumin across GEnC monolayers when exposed to H_2_O_2_ at 100 µM. This effect can be partially blocked when GEnC were pre-treated with free radical scavengers, catalase and superoxide dismutase. (n = 11, p<0.05 by 2 way ANOVA; control vs H_2_O_2_100 µM, and H_2_O_2_100 µM vs H_2_O_2_100 µM+block using bonferroni analysis).

In the light of the findings on TEER described above and to avoid a confounding effect of reduced TEER (suggesting increased junctional permeability), estimation of albumin passage across GEnC monolayers was performed after 120 min of exposure. H_2_O_2_ significantly increased passage of albumin across GEnC monolayers in a dose dependant manner (Figure 7A). The effect of H2O2 treatment was significant at 100 µM and 200 µM. In a set of separate experiments, the effect of H_2_O_2_ at 100 µM could be partially blocked with free radical scavengers, superoxide dismutase and catalase (Figure 7B).

## Discussion

In our study we used human GEnC that are conditionally immortalized and express the typical phenotypic characteristics. These cells have been well studied previously and express glycocalyx under culture conditions [12], [14]. There were no significant changes in GEnC survival after exposure to H_2_O_2_ as judged by production of formazan using the WST-1 assay. GEnC were also carefully monitored by phase contrast microscopy during experiments and no significant problems with monolayer formation or cell detachment were noted at the concentrations of H_2_O_2_ used for treatments in experiments. Our results demonstrate loss of glycocalyx from the surface of GEnC after exposure to H_2_O_2_. This was evident from significant loss of WGA lectin and HS immunostaining from the surface of GEnC. The loss of GAG from GEnC cell surface was associated with markedly increased HS GAG fractions in the supernatant. This was first demonstrated using an Alcian Blue colorimetric assay (indicating loss of anionic residues) and then upon quantification of radiolabelled GAG fractions separated by liquid chromatography. Analysis of incorporated radiolabeled 3H-glucosamine and S^35^ was used to study biosynthesis of GAG over 48 h after treatment with H_2_O_2_. Our results did not show significant changes in the biosynthesis of either total or sulphated GAG chains after exposure to of H_2_O_2_ over a 48 h period of analysis. The effect of ROS is short-lived and the later finding confirms their direct action on the sulphated GAG as suggested by the Alcian blue binding experiments. ROS reduced the TEER of GEnC monolayers and increased the paracellular transport. The effect was immediate, lasting for 90 min with maximal effect at 30 min. The relatively short term and reversible effect of ROS (particularly H_2_O_2_) has also been previously reported [24] and is due to increased gaps in the endothelial junctions [25] and mediated *via* Ca^+^ signaling pathways [26]. ROS caused an increase in the macromolecular passage in a dose dependant manner. The changes observed showing cleavage of HS after ROS coupled with increase in macromolecular passage follow a similar pattern seen after treatment with HS degrading enzymes reported previously [14]. It is therefore highly probable that loss of HS from GEnC glycocalyx after to ROS is likely responsible for the increase in macromolecular passage observed in the FITC-albumin experiments. It was also reassuring to see that effect of ROS could be partly inhibited by free radical scavengers.

ROS are well known to depolymerise and damage the structure of HS, CS and HA GAG [27]. Non-sulphated GAG, HA appears to be particularly susceptible to damage from ROS [28]. Loss of sulphation from GAG chains particularly HS is a known feature in diabetes and is linked to chronic hyperglycemia [29]. This line of evidence suggests that the changes in the chemical structure of GAG, particularly loss of sulphation may render the GEnC glycocalyx more susceptible to oxidant damage. Interestingly, our results also show that ROS largely cleaves sulphated GAG (HS) from the GEnC glycocalyx.

H_2_O_2_ is a major source of ROS in the microvascular endothelium and is widely used to model oxidative stress *in vitro* and *in vivo* [30], [31]. H_2_O_2_ injected directly into the renal artery causes reversible nephrotic range proteinuria in an experimental model without any evidence of ultrastructural abnormality in the GFB [32]. Similarly, ROS have been shown to cause a defect in both size and charge selectivity of the GFB with an intact podocyte and GBM structure in an ischaemia-reperfusion injury model [33]. Onset of proteinuria following generation of oxidative stress in mouse glomeruli has also been associated with increased excretion of HS GAG. Both proteinuria and loss of glomerular HS GAG could be reversed upon blockade of ROS [34]. Indeed, hydroxyl radicals generated by H_2_O_2_ are also known to depolymerise HS GAG [35]. In the absence of direct experimental investigation, the evidence above suggests a role of GEnC glycocalyx in the dysfunction of the GFB caused by oxidant injury.

The results of our study suggest that ROS disrupt GEnC glycocalyx through a direct mechanism of action without affecting the GAG biosynthetic pathway. This is the first study that elucidates a potential mechanism by which ROS can disrupt the barrier properties of GEnC glycocalyx. These findings suggest that GEnC glycocalyx plays a significant role in the pathogenesis of oxidant induced glomerular injury. Excessive generation of ROS is associated with the pathogenesis of a number of glomerular diseases including diabetic nephropathy [36] and acute kidney injury due to ischemia-reperfusion injury and sepsis [37]–[39]. Future efforts should be focused to dissect the molecular pathways in order to develop new molecular therapeutic targets.

## Materials and Methods

### GEnC Culture

We used a normal human conditionally immortalised GEnC line as described in detail previously [12]. Briefly, primary culture GEnC were exposed to separate retroviral vectors transducing a temperature sensitive mutant of SV40 large T antigen and the catalytic subunit of human telomerase. At the permissive temperature of 33°C the tsSV40LT transgene is activated causing cell proliferation (without telomere shortening) while at 37°C the transgene is inactivated rendering cells non-proliferative and quiescent. Conditionally immortalised GEnC were used for experiments after maintaining them at the non-permissive temperature for 7 days. GEnC were cultured in endothelial growth medium-2 microvascular (EGM2-MV, Cambrex, Wokingham, UK) containing foetal calf serum (5%) and growth factors as supplied with the exception of VEGF.

### Generation of ROS and GEnC Survival Assay

H_2_O_2_ (Sigma-Aldrich Cat: 516813) was used at concentrations from 50–500 µM in preliminary experiments to determine cellular toxicity. H_2_O_2_ and hydroxyl radical (OH-) acts as ROS. This was assessed by phase contrast microscopy. Further, a cell survival assay was performed using a commercially available WST-1 assay kit (Roche, Germany). For the WST-1 assay, GEnC were incubated with 1∶10 dilution of WST-1 reagent for 2 h. 200 µl of media from each well was pipetted into a 96 well plate and analysed on a spectrophotometer at 420–480 nm.

100 µM of H_2_O_2_ was taken as the optimal concentration (no evidence of cell toxicity) for all experiments:‘ROS media’. SOD (from bovine erythrocytes; Sigma-Aldrich S25115) and catalase (from bovine liver; Sigma-Aldrich C1345) were used to scavenge ROS. The powder form was solubilized in serum free media and used at 300 U/ml and 1200 U/ml respectively. For inhibition of ROS: both SOD and catalase were added at the same time as 100 µM of H_2_O_2_. These concentrations were derived from a previously published protocol [24].

### Metabolic Labeling and Extraction of Proteoglycans

Metabolic labeling of GAG was achieved by culturing GEnC in media containing 50 µCi/ml [S^35^] sulfate and 20 µCi/ml D- [3H^3^] glucosamine (Amersham Biosciences). After 10 days, the media in the ‘control’ GEnC was replaced by serum free media (vehicle) whereas the test GEnC media was changed to ROS media (100 µM of H_2_O_2_). Next, S^35^-sulphate and 3H^3^-glucosamine was added to the respective media for 48 h. Medium was collected and filtrated through a 0.22 µm filter (Millipore, MA). Cells were extracted in RIPA lysis buffer containing 10% (v/v) Triton X-100, 10% (m/v) SDS, 10% (m/v) sodium deoxycholate and Complete Mini Protease Inhibitor (Roche, Germany) in phosphate buffered saline and filtrated through a 0.22 µm filter. Protein concentration was measured using a BCA protein assay kit (Pierce Labs, IL). For radiolabelling studies to estimate cleavage of GAG in the supernatant, cells were treated with 20 uCi/ml of D-3H^3^ glucosamine and incubated for 24 hours. The media was then removed and cells gently washed in PBS. Serum free media was then added, containing 100 uM H_2_O_2_ for 2 h. Media was then collected and concentrated using a 3 kDa molecular weight cut off Millipore filter and run on the Anion Exchange Liquid Chromatography to isolate fractions.

### Isolation of Proteoglycans

A HiTrap^®^ DEAE FF (1 ml; Amersham Biosciences) column connected to a ÄKTA™ FPLC system (Amersham Biosciences) was equilibrated with 6 M urea, 0.5 M NaOAc, pH 5.8, 5 µg/ml bovine albumin, 0.1% Triton X-100 (equilibration buffer). Sample was applied to the column at 1 ml/min and subsequently merged with column matrix for 15 min. The column was then washed successively with 10 ml of three different buffers, at 10 ml/min. 1) Equilibration buffer, 2) 6 M urea, 10 mM Tris, pH 8.0, 5 µg/ml bovine albumin, 0.2% Triton X-100; and 3) 50 mM Tris pH 7.5. The bound proteoglycans were eluted with 4 M guanidine-HCl, 50 mM NaOAc, pH 5.8, 5 µg/ml bovine albumin, 0.2% Triton X-100 and collected in 1 ml fractions. All fractions were quantified by liquid scintillation counting on a Beta Counter LS6500 (Beckman Coulter) with Ready Safe cocktail (Beckman Coulter).

### Immunofluorescence and Lectin Binding

Cells were seeded on glass cover slips and cultured for 24 h before exposing to either control or ROS media for 48 h, and fixed in 2% paraformaldehyde for 10 min. Coverslips were then incubated either in blocking solution (5% FCS and 0.05% Tween20 in PBS) followed by antibody incubation for HS (1∶500; HepSS-1, US Biologicals, Swampscott, MA) or directly with FITC-conjugated wheat germ agglutinin (WGA, from Triticum vulgaris) lectin (Sigma-Aldrich, St. Louis, MO) at 2 µl/ml for 30 min. WGA (binds to the carbohydrate moieties N-acetyl glucosamine and N-acetyl neuraminic acid on glycocalyx constituents). Nuclear staining was demonstrated using 4,6-diamidino-2-phenylindole (DAPI). Coverslips were examined using a Leitz DMRB fluorescence microscope (Leica, Solms, Germany). Fluorescence intensity was quantified using a fluorescence plate reader as previously described [14].

### Alcian Blue Dye Binding Assay for Quantitation of Sulphated GAG

GEnC were treated with 100 µM H_2_O_2_ or vehicle in serum free media for 1 h. Conditioned media were collected and precipitated in 95% ethanol containing 1% potassium acetate. After centrifugation at 500 g and 4 degree C for 10 min the pellet was resuspended in 100 µl PBS. A 50 µl sample was incubated with 200 µl alcian blue solution for 15 min at room temperature. Absorbance was then read at 490 nm. The ionic interaction between the cationic dye (Alcian blue) and the negatively charged GAG is proportional to the number of negative charges present. The concentration of sulphated GAGs in the samples was quantitated using a chondroitin sulphate standard curve (0.02–0.313 mg/ml).

### Measurement of Trans-endothelial Electrical Resistance

Trans-endothelial Electrical Resistance (TEER) was measured using an automated bioimpedance sensing system (ECIS™; Applied Biophysics Inc, NY) as described previously [14]. GEnC were seeded in 8 well, 10 electrode per well arrays (8W10E) supplied by the manufacturer. 100 µM of H_2_O_2_ was added to the treatment wells at time point zero. The resistance is reported in Ω and the measurement from each well, at a given time point, is an average from 10 electrodes.

### Measurement of Transendothelial Protein Passage

GEnC were seeded on polycarbonate supports (0.4 µm pore size, 0.5 cm^2^ surface area) in tissue-culture inserts (1 cm diameter; Nalge Nunc International, Rochester, NY) and treated with ROS or control media as above for 2 h prior to measuring passage of FITC-labeled bovine serum albumin (BSA, Sigma) across the monolayer as described previously [14]. Briefly, the medium in the insert was replaced with 500 µl of SFM containing 0.5 mg/ml FITC-BSA; that in the well was replaced with 500 µl of SFM containing 0.5 mg/ml unlabeled BSA (Sigma). At 1, 2, and 3 h, 100 µl aliquots were removed and replaced with 100 µl of SFM containing unlabeled BSA (0.5 mg/ml). The fluorescence of the aliquots was measured as above, and the cumulative amount of FITC-BSA passing through the monolayer was calculated by reference to a set of standard dilutions.

### Statistical Analyses

Graph Pad Prism-4 statistical software package (Graph Pad Software Inc. San Diego, CA) was used for all analyses, including standard error of mean (SEM) t-tests and ANOVA. P<0.05 was taken to indicate statistical significance.
